# A comparison of arterial spin labeling and dynamic susceptibility perfusion imaging for resection control in glioblastoma surgery

**DOI:** 10.18632/oncotarget.24970

**Published:** 2018-04-06

**Authors:** Thomas Lindner, Hajrullah Ahmeti, Julia Juhasz, Michael Helle, Olav Jansen, Michael Synowitz, Stephan Ulmer

**Affiliations:** ^1^ Clinic for Radiology and Neuroradiology, UKSH Kiel, Kiel, Germany; ^2^ Clinic for Neurosurgery, UKSH Kiel, Kiel, Germany; ^3^ Philips Research Laboratories, Hamburg, Germany

**Keywords:** magnetic resonance, imaging, perfusion, glioblastoma, in-vivo

## Abstract

Resection control using magnetic resonance imaging during neurosurgical interventions increases confidence regarding the extent of tumor removal already during the procedure. In addition to morphological imaging, functional information such as perfusion might become an important marker of the presence and extent of residual tumor mass. The aim of this study was to implement arterial spin labeling (ASL) perfusion imaging as a noninvasive alternative to dynamic susceptibility contrast (DSC) perfusion imaging in patients suffering from intra-axial tumors for resection control already during surgery. The study included 15 patients suffering from glioblastoma multiforme in whom perfusion imaging using DSC and ASL was performed before, during, and after surgery. The data obtained from intraoperative scanning were analyzed by two readers blinded to any clinical information, and the presence of residual tumor mass was evaluated using a ranking scale. Similarity of results was analyzed using the intraclass correlation coefficient and Pearson's correlation coefficient. The results show that intraoperative ASL is as reliable as DSC when performing intraoperative perfusion imaging. According to the results of this study, intraoperative imaging using ASL represents an attractive alternative to contrast agent-based perfusion imaging.

## INTRODUCTION

Magnetic resonance imaging (MRI) is considered the gold standard for perioperative imaging of brain tumors because of its capability to acquire anatomical and pathological structures with a spatial resolution of less than one millimeter and with an unchallenged soft-tissue contrast without exposing the patient to ionizing radiation. During (neuro-)surgical interventions MRI helps further to achieve the goal of maximal resection while sparing non-infiltrated tissue. Therefore, obtaining as much information as possible is crucial, especially when “eloquent” brain regions are at risk. Maximum removal of a tumor has been found to be one of the prognostic factors for progression-free and overall survival [1, 2].

The invasive nature of surgery on the brain surface and the deeper lying tissue temporarily disrupts the blood-brain barrier and may cause accumulation of contrast agents, which can be seen on intraoperative MR images at the rim of the resection cavity. This enhancement might not be distinguishable from residual tumor using conventional anatomical imaging only [3, 4]. For this reason, intraoperative perfusion imaging was introduced and has proven to be a reliable method of identifying residual tumor during surgery and distinguishing it from surgically induced contrast enhancement [3, 5].

Dynamic susceptibility-weighted (DSC) MRI is a commonly used perfusion technique that is based on the intravenous administration of contrast agent (CA). Images are acquired using rapid T2* imaging while the CA passes through the brain tissue. Using DSC-MRI, several parameters are commonly assessed [6–9]. Cerebral blood flow (CBF) in DSC is defined as the quotient of cerebral blood volume (CBV) and the mean transit time (MTT) of the CA passing through the tissue [10–12]. The use of CA, however, represents one major disadvantage of this method, as it limits the number of times measurements can be repeated and can pose a problem in patients who might have allergic reactions or impaired renal function, potentially leading to nephrogenic system fibrosis (NSF). Recent findings also suggest that some by-products of CAs accumulate inside the brain parenchyma [13–15]. It is important to note that some patients need to undergo three MRI examinations with CA routinely on three consecutive days (i.e., for planning and intra- and postoperative scanning). Therefore, any reduction in CA dose should be welcomed.

One alternative method of acquiring information about perfusion without administering any CA is arterial spin labeling (ASL) [16]. In ASL, the perfusion-weighted images are obtained by subtracting two acquired datasets in which the magnetic state of blood differs, i.e., one image is performed with inversion of inflowing blood spins (label), while the other image is performed without (control). As the signal of static tissue remains unchanged, the subtraction image yields the difference in blood magnetization in gray matter [17]. After labeling the blood, a certain delay in time (postlabeling delay, PLD) must be awaited until the tagged blood reaches the capillary bed of the tissue [16]. During this time, the inverted blood spins inevitably begin to relax according to their specific constant of T1, continuously decreasing the signal difference between the label and control image, requiring scan times of approximately 3–4 minutes [17]. Due to longer T1 relaxation constants, ASL benefits from higher magnetic field strengths (e.g., 3T), but can be performed on 1.5T scanners as well [16, 18]. One major advantage of ASL is the option to quantify perfusion in absolute values (in ml/min/100 g brain tissue) using mathematical models, which is not possible using any contrast-enhanced perfusion technique [11].

Due to the limitations of DSC perfusion imaging and the potential of ASL, the aim of this study is to evaluate ASL imaging as an alternative method for neurosurgical resection control in direct comparison to DSC perfusion imaging, the latter being considered the gold standard method.

## RESULTS

### Magnetic resonance imaging

Data acquisition was successfully performed in all but one patient. In this one case the intraoperative DSC acquisition could not be successfully acquired due to a technical error shortly after contrast agent administration. As the scan could not be started due to an activated interlock of the MRI door, the passage of the bolus could not be visualized. A second application of CA was waived by the neurosurgeon. Therefore, this patient had to be excluded from further analysis.

**Figure 1:**
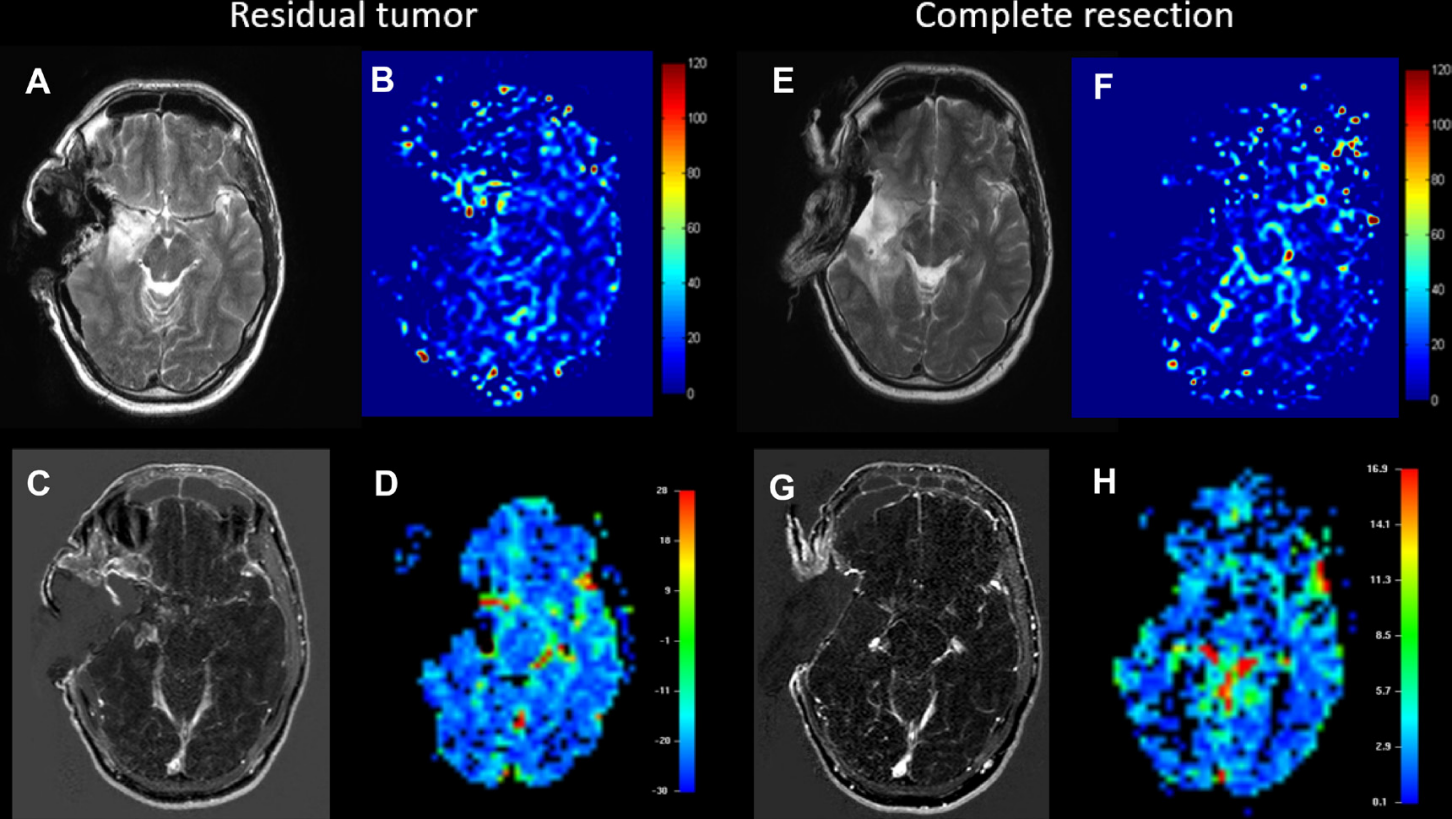
Example of a patient with residual tumor (**A**–**D**) and a case of complete resection of the glioblastoma (**E**–**H**). A and E show T2-weighted images serving as anatomical reference for the ASL, which is shown in B and F (CBF is displayed in 100 ml/min/100 g tissue). C and G show contrast-enhanced T1-weighted images and finally D and H show negative integral maps of the DSC perfusion (Colormap in arbitrary units). All images were performed using the 1.5T intraoperative scanner.

Two representative examples in which image acquisition worked properly are shown in Figure 1. In the first case (residual tumor) an incomplete resection is shown. In this case, the surgeon could not remove the tumor mass completely, leaving an area of elevated perfusion (i.e. residual tumor) in the central area. This can be seen on both the DSC as well as the ASL (Figure 1B and 1D). The second case shows complete resection of a right-hemispheric tumor (Figure 1F and 1H). Neither in the ASL nor in the DSC residual tumor mass can be detected, indicating removal of all areas of elevated perfusion. (Figure 1A and 1C) show T1 images of the first patient with and without contrast agent and (Figure 1E and 1G) for the second respectively. The follow-up (not shown) also showed that no residual tumor growth could be detected.

**Figure 2:**
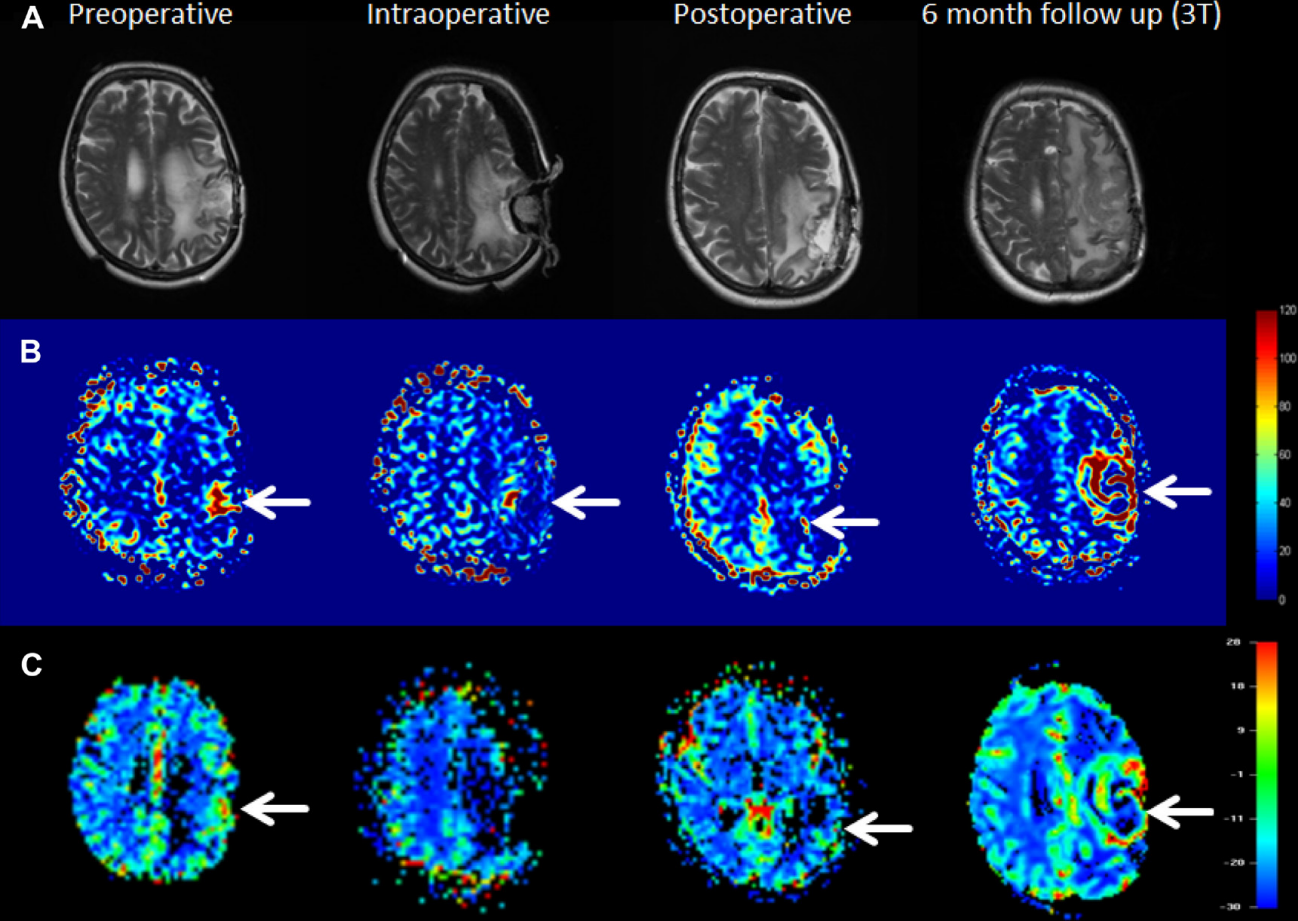
Example of a patient with incomplete resection and following residual tumor growth at the 6-month follow-up examination In the upper row (**A**) anatomical images are shown. The second row (**B**) shows ASL data in which residual tumor can be clearly seen in all time points and the last row (**C**) show DSC data with visualization of elevated perfusion in all cases except the intraoperative image acquisition, which might be attributed to the low signal-to-noise ratio in the intraoperative setting. All three images around the surgical intervention were acquired on 1.5T except the 6-month follow-up image, which was acquired using 3T.

Another case is shown in Figure 2. In this case a discrepancy between ASL and DSC became evident as ASL showed residual tumor already during surgery which was however not visible in the DSC images. Irrespective the results from imaging, no further resection was being performed due to the proximity of eloquent areas. In the post-operative examination, both ASL and DSC showed elevated perfusion adjacent to resection cavity. In the 6-month follow up imaging the residual tumor grew rapidly, infiltrating a larger volume of the left hemisphere.

### Statistical analysis

The intraclass correlation coefficient between the two readers was 0.93 and 0.98 for ASL and DSC, respectively, indicating excellent correlation between the two. Following this result, both image quality and reliability of these techniques appear well suited to be used in this setting.

The Pearson's correlation was 0.66 and 0.69 for reader 1 and 2, respectively, indicating a moderately positive relationship between the scanning methods. Albeit this positive relationship, upon in-depth evaluation of the results, it appears that ASL is better suited to detect residual tumor mass as in one case both readers did no see residual tumor in the DSC. Regarding the depiction whether tumor was clearly visible (rating score 2) or unclear (rating score 1), no method appears better than the other, as these results were in most cases identical.

## DISCUSSION

In this study, we assessed the feasibility of ASL perfusion imaging as an alternative to DSC perfusion imaging for resection control during neurosurgical interventions. Resection control using MRI in a neurosurgical setting is commonly performed using T2w and native and contrast-enhanced T1w morphological imaging. In some cases, morphological imaging might be limited, hampered or even unlabeled, e.g., by a surgically induced temporary disrupted blood-brain-barrier, which leads to false-positive contrast enhancement of the resection margin and can thus impose itself as contrast-enhancing residual tumor tissue [22]. To compensate for this, intraoperative perfusion imaging using DSC (sometimes also dynamic contrast-enhanced, DCE) MRI was introduced. This method is based on applying CA intravenously and then tracking the bolus passage through the tissue. DSC-MRI intraoperatively has already proven to distinguish surgically induced artificial enhancement intraoperatively from remaining tumor tissue [3]. The functional information provided by perfusion imaging allows for deeper insights into pathophysiology and conclusions can be drawn about adjacent tissue next to the resection cavity. ASL seems to been a reliable alternative. A recent study already evaluated ASL and anatomical imaging in direct comparison: here, ASL provided more accurate results than structural imaging only in a small patient cohort [19]. Another study evaluated the effects of anesthesia using ASL [20]. The latter could also become more interesting regarding changes of perfusion of tumors under anesthesia.

The obtained results from this study indicate that ASL and DSC do not perfectly correlate in their potential to distinguish residual tumor during surgery Detailed results are shown in Table 2. A Pearson's correlation of only 0.66 and 0.69 for the readers indicates only moderate correlation of the two methods. Upon further inspection of the results, a small advantage for ASL can be observed regarding the clarity of visualized lesions, as both readers gave higher mean scores for ASL than for DSC. In no case has DSC proven better than ASL.

**Table 1:** Detailed data about the study patients

Patient	Age (years)	Primary (P)/Recurrent (R)	Preoperative DSC (Days to surgery)	Preoperative ASL (Device/days to surgery)	Postoperative ASL and DSC (Device/days after surgery)
1	61	P	+1	1.5T/+1	1.5T/+1
2	47	R	+5	1.5T/+1	1.5T/+1
3	51	P	+3	iOP/0	1.5T/+3
4	49	R	+3	iOP/0	1.5T/+1
5	50	R	+1	1.5T/+1	1.5T/+3
6^*^	51	P	+5	1.5T/+1	1.5T/+3
7	47	R	+1	iOP/0	1.5T/+1
8	55	R	+1	iOP/0	1.5T/+1
9	46	P	+3	1.5T/+1	1.5T/+1
10	47	P	+1	1.5T/+1	1.5T/+1
11	44	R	+2	1.5T/+1	1.5T/+1
12	48	P	+1	1.5T/+1	1.5T/+3
13	53	R	+1	iOP/0	1.5T/+1
14	55	R	+3	1.5T/+1	1.5T/+1
15	61	R	+2	iOP/0	1.5T/+1

The devices used were the 1.5T and 3T from the radiology department and iOP is the scanner in the OR. A distinction between primary (P) and recurrent (R) disease is also indicated.

^*^This patient was excluded from further statistical analysis as the DSC scan could not be acquired successfully.

**Table 2:** Individual ratings of the readers from the intraoperative images, where 0 means no residual tumor mass, 1 describes an unclear lesion/unsure diagnosis, and 2 definitely indicates residual tumor mass

Patient	Rater 1		Rater 2	
1	ASL	DSC	ASL	DSC
1	2	2	2	2
2	0	0	0	0
3	2	2	2	2
4	1	1	1	1
5	2	2	2	2
6^*^	2	—	2	—
7	2	0	2	0
8	1	1	1	1
9	2	2	1	2
10	2	1	2	2
11	0	0	0	0
12	2	2	1	1
13	1	2	1	2
14	0	0	0	0
15	2	1	2	2

^*^This patient was excluded from further statistical analysis as the DSC scan could not be acquired successfully.

The ICC, on the other hand, shows excellent reliability of > 0.9 for both methods, indicating that the two readers agreed as to whether tumor was present or not (proven postoperatively in follow-up imaging).

ASL perfusion holds several advantages over DSC. First, ASL is completely noninvasive, meaning that no external substances need to be administered. Some patients undergo three MR examinations within three consecutive days (pre-, intra-, and postoperative MRI) and in each examination, CA is administered. This dose, which is applied repeatedly in a short period of time, can cause accumulation of the agent in the brain tissue [15]. Secondly, due to its noninvasive nature, ASL can be repeated without adverse effects if the acquisition did not work properly the first time, e.g., the PLD was set too low for the patients’ arterial transit time and the labeled bolus has not yet arrived in the tissue, leading to false assumptions of CBF. If, on the other hand, the DSC acquisition is hampered (e.g., technical defects, patient movement, etc.) or the bolus was administered too early and could not be caught during the acquisition, repeated acquisitions might become problematic due to the presence of already administered CA. This was the case in one patient in this study. Another advantage of ASL is that adjacent vessels do not influence the results of the images as the PLD is set to a time that is longer than the arterial transit time in the patients and therefore all (tagged) blood has already been delivered to the tissue at the time of readout [16]. Still, under some conditions, e.g., blood vessel occlusion or in deep anesthesia, blood flow might become severely reduced, leading to remaining signal inside the (major) intracranial arteries only and potentially leading to misinterpretation [23], which, however, would also hamper results from DSC-MRI Additionally, the contrast-to-noise-ratio (CNR) of ASL can be easily adjusted by increasing the number of times the scan is repeated. Performing more repetitions increases CNR but also scan time. This is not the case when performing DSC as the CNR is mainly influenced by the applied CA, so there is no other option to increase CNR in DSC-MRI.

Currently, ASL perfusion can only be quantified accurately in terms of ml blood/100g/min by using single-PLD ASL [16]. Multi-PLD ASL has been proposed in recent years and used to extract multiple parameters that were only assessed by DSC [24, 25]. Accurate quantification of multi-PLD ASL data is an ongoing topic in research and should therefore be considered in future studies to directly compare the multifactorial data of DSC and ASL. Generally, ASL and especially multi-PLD ASL acquisitions need a few minutes longer in scanning than DSC, which should be no problem in the OR for improvement of the information gained by this technology, the option to repeat it several times if necessary and the advantage to avoid any contrast agent. Longer scan durations, however, can become a severe limitation in pre- and postoperative scanning, especially in patients whose cognitive functions are impaired due to the tumor or in patients, who are in pain or claustrophobic, causing them to move their head during acquisition.

To conclude, the use of ASL appears attractive as an alternative method of resection control by means of perfusion already during surgery. The results from this study indicate only a moderate correlation to the gold standard of DSC MRI, but these results are in favor of the ASL technique. Future studies using multi-PLD ASL methods could further increase our knowledge about the specific disease of a patient and disease progression, ultimately increasing confidence regarding outcome in these individuals.

## MATERIALS AND METHODS

### Study population and data acquisition

In this study, 15 consecutive patients (11 men, 4 women, mean age: 51.4 ± 5.2 years) suffering from glioblastoma multiforme (GBM) presenting either as primary or recurrent disease were included. Data were collected according to the policies of the local ethics committee. All patients underwent preoperative MRI for diagnostic purposes and surgical planning, intraoperative MRI for resection control, and postoperative MRI for the evaluation of surgical success after written informed consent. Inclusion criteria were general MRI compatibility of the patients, the consecutive scans must not be longer than three days apart (e.g., if surgery was scheduled on a Friday, the postoperative scan was performed on Monday), and DSC perfusion must not be older than 1 week prior to surgery.

A detailed list of the patients’ data can be found in Table 1.

### Magnetic resonance imaging

Imaging was performed for each patient on two or three MRI scanners (all from Philips Healthcare, Best, The Netherlands), including a 3T Achieva scanner with a 32-channel head coil, denoted as “3T”, and a 1.5T Achieva scanner with a 6-channel head coil, denoted as “1.5T”, for pre- and postoperative imaging, as well as a 1.5T Intera scanner equipped with two one-channel circular coils in the operating room (OR), denoted as “iOP”. A recent study evaluated the comparability and reproducibility of ASL data using these scanners in volunteers and showed initial, positive results in a small patient study [19]. Pre- and postoperative imaging were performed on the 1.5T or 3T (or both) scanners, depending on the clinical routine schedule for the equipment. Intraoperative scanning was performed solely on the iOP system. In some cases preoperative ASL was also performed using the iOP directly before surgery but already after anesthesia, e.g., when the ASL scan was not performed during primary diagnostic scanning. A list showing which scanner was used for each patient is shown in Table 1.

DSC perfusion imaging was performed on each scanner as a 2D multislice echo-planar (EPI) sequence with a 3.44 × 3.44 mm^2^ voxel size and 30 slices with a thickness of 3.5 mm (TR/TE: 25/17, EPI factor: 17, field-of-view: 230 × 230 × 105 mm, flip angle: 7°), and 60 dynamic scans.

ASL was performed on all scanners using pseudo-continuous ASL (pCASL) with a labeling duration and a PLD of 1800 ms. Image readout was performed using EPI imaging with a 3.6 × 3.5 mm^2^ voxel size and 16 slices with a thickness of 5 mm (TR/TE: 2616/13, EPI factor: 39, field-of-view: 240 × 240 × 95 mm, flip angle: 90°). The number of label/control pairs was 20 at 3T, 30 at 1.5T, and 40 in the OR to account for reduced signal due to the weaker magnetic field and reduced number of receive channels [19].

Additionally, a T2-weighted turbo spin echo sequence was acquired with the same slice thickness as the ASL scan to serve as anatomical reference. Scan parameters were: TR/TE 1858/80, TSE factor 19, 2.7 × 2.7 mm^2^ voxel size, and 16 slices with 5 mm thickness; flip angle: 90°.

### Image processing and statistical analysis

All DSC perfusion data were evaluated using the Philips View Forum platform (Philips Healthcare, Best, The Netherlands). From the acquired data, maps of the negative integral, T0, time-to-peak (TTP), mean transit time (MTT), and index map were calculated routinely. For analysis, the negative integral was mainly used, the remaining maps only in unclear cases.

The ASL data were postprocessed using a Matlab Routine (Matlab R2013b, The Mathworks, Natick, MA) developed in-house, first to calculate relative CBF maps by subtracting the label and control data and further to calculate quantitative CBF maps (expressed in ml/min/100 g) using the algorithm suggested in [16].

The presence of residual tumor was assessed using a 3-point rating scale, in which 0 means no visible residual tumor mass, 1 describes an unclear lesion/uncertain diagnosis, and 2 definitely indicates residual tumor mass.

The readers were allowed to use the preoperatively acquired DSC and ASL data, which served as information about tumor size, location, and perfusion signal, but they were blinded to other images with or without contrast agent and also to clinical information regarding the extent of the resection from the surgeon.

The intraclass correlation coefficient (ICC) was calculated to evaluate the inter-rater agreement between the two readers in identifying residual tumor (0.0–0.5 = poor reliability; 0.5–0.75 = moderate reliability; 0.75–0.90 = good reliability; 0.90–1.0 = excellent reliability) [21].

The Pearson's correlation coefficient was calculated to evaluate the similarity of the results obtained by DSC and ASL for each reader, i.e., if residual tumor was visible or not.

## Abbreviations

ASL: Arterial Spin Labeling
CA: Contrast Agent
CBF: Cerebral Blood Flow
CBV: Cerebral Blood Volume
CNR: Contrast to Noise Ratio
DCE: Dynamic Contrast Enhanced
DSC: Dynamic Susceptibility Contrast
EPI: Eco Planar Imaging
GBM: Glioblastoma Multiforme
ICC: Intra Class Correlation Coefficient
MRI: Magnetic Resonance Imaging
MTT: Mean Transit Time
OR: Operating Room
pCASL: pseudo continuous Arterial Spin Labeling
PLD: Post Labeling Delay
TTP: Time to Peak.
